# Global School-Based Childhood Obesity Interventions: A Review

**DOI:** 10.3390/ijerph110908940

**Published:** 2014-08-28

**Authors:** Melinda J. Ickes, Jennifer McMullen, Taj Haider, Manoj Sharma

**Affiliations:** 1 Department of Kinesiology and Health Promotion, College of Education, University of Kentucky, Lexington, KY 40506, USA; E-Mail: jmc255@g.uky.edu; 2 The Legal Aid Society, 199 Water Street, New York, NY 10038, USA; E-Mail: thaider@legal-aid.org; 3 Behavioral & Environmental Health, Jackson State University, Jackson, MS 39213, USA; E-Mail: manoj.sharma@jsums.edu

**Keywords:** obese, overweight, school-based, youth, child, prevention, intervention, program

## Abstract

*Background*: The issue of childhood overweight and obesity has become a global public health crisis. School-based interventions have been developed and implemented to combat this growing concern. The purpose of this review is to compare and contrast U.S. and international school-based obesity prevention interventions and highlight efficacious strategies. *Methods*: A systematic literature review was conducted utilizing five relevant databases. Inclusion criteria were: (1) primary research; (2) overweight or obesity prevention interventions; (3) school-based; (4) studies published between 1 January 2002 through 31 December 2013; (5) published in the English language; (6) child-based interventions, which could include parents; and (7) studies that reported outcome data. *Results*: A total of 20 interventions met the inclusion criteria. Ten interventions each were implemented in the U.S. and internationally. International interventions only targeted elementary-aged students, were less likely to target low-income populations, and were less likely to be implemented for two or more years in duration. However, they were more likely to integrate an environmental component when compared to U.S. interventions. *Discussion*: Interventions implemented in the U.S. and internationally resulted in successful outcomes, including positive changes in student BMI. Yet, varying approaches were used to achieve success, reinforcing the fact that a one-size-fits-all approach is not necessary to impact childhood obesity. However, building on successful interventions, future school-based obesity prevention interventions should integrate culturally specific intervention strategies, aim to incorporate an environmental component, and include parents whenever possible. Consideration should be given to the potential impact of long-term, frequent dosage interventions, and subsequent follow-up should be given attention to determine long-term efficacy.

## 1. Introduction

Obesity continues to threaten health outcomes and quality of life worldwide, particularly among youth. Obesity, once a problem specific to nations of wealth, now impacts, to varying degree, countries of all economic levels [1]. Overall, global obesity rates are higher in adults than children. However, in the U.S., Brazil, China, and other countries, the epidemic has increased at a faster rate in children than in adults [2]. As indicated in previous studies, the incidence of overweight and obesity differs globally [3]. Childhood overweight and obesity have increased more dramatically in economically developed countries and in urbanized populations [3]. According to a 2006 study of worldwide childhood obesity trends, the prevalence of obesity in school-aged children was the following: Africa, 0.2%; Americas, 9.6%; Eastern Mediterranean, 5.9%; Europe, 5.4%; South East Asia, 1.5%; and West Pacific, 2.3% [3]. The prevalence of global childhood overweight and obesity increased from 4.2% in 1990 to 6.7% in 2010, with a total of 43 million children estimated to be overweight or obese in 2010, including 35 million in developing countries. Trends estimate that in 2020 the rates of global childhood overweight and obesity will increase from 6.7% to 9.1% [4]. Although a concern worldwide, prevalence rates in the United States tend to be higher as compared to other developed countries [5]. The obesity rates for children ages 2–19 in the United States from 1980 to 2010 have more than tripled [6]. The most recent National Health and Nutrition Examination Survey (NHANES) indicated that 16.9% of 2–19 year olds in the United States were obese and 31.7% were overweight or obese. Specifically, the prevalence of obesity in 2009–2010 was 12.1% among children ages 2–5, 18.0% among children ages 6–11, and 18.4% among children ages 12–19 [7]. While obesity remains a global concern, recent findings indicate a plateau in the spiked increases observed over the past 30 years and in some cases a marked decline in several developed countries [5]. As stated by Olds and colleagues [5], “While rates of overweight and obesity appear to be stabilizing at present in many countries, they are still unacceptably high, with significant ramifications for the health and well-being of these children as they age (p. 355, [5]). As a result, the need to determine effective strategies to prevent and mitigate overweight and obesity is still urgent.

Obesity has a number of health, social, and economic consequences. From an economic viewpoint, obesity places a strain on the healthcare system [8]. In fact, in a recent review of the economic burden of obesity worldwide, individuals struggling with obesity have medical costs 30% greater than those of normal weight [8]. Considering the individual impact, childhood obesity has been associated with a number of negative secondary health-related outcomes. Obese children are more likely to have increased blood pressure and increased cholesterol levels—both of which are risk factors for cardiovascular disease [9]. Childhood obesity also increases the likelihood of insulin resistance and glucose intolerance, leading to diabetes mellitus type 2 [10]. In addition, negative psychological and emotional outcomes have been reported, including low self-esteem and body-esteem, depression, and stigmatization [11]. Moreover, obese children are more likely to become severely obese adults–further necessitating the need to prioritize prevention efforts [9].

Obesity is shaped by a number of determinants: common genetic variants, influences within the first year of life, maternal behaviors, family food environment and dietary behaviors, physical activity and inactivity, and environmental factors that either hinder or enhance one’s accessibility to healthy food and physical activity [12,13,14,15,16]. Not only should childhood obesity prevention interventions aim to target modifiable determinants of obesity, but the setting in which these interventions are implemented also needs to be considered to truly impact this global epidemic.

School-based programs have historically been used to impact child health. Specifically, preventive efforts targeting childhood obesity have frequently focused on schools as an important setting [12,16]. Schools have been considered an ideal target, given the propensity to prevent obesity through the promotion of physical activity, nutritious food offerings, and nutrition education through practice, policy, and supportive environments [12,16]. Targeting school-aged children is logical given that physical activity and dietary habits are imprinted at this age, allowing schools the opportunity to establish life-long healthy habits in children [14]. Past systematic reviews have highlighted the success of school-based obesity prevention interventions [12,17,18,19]. Implications from a review incorporating interventions among children between 1966 and 2001 indicated there were minimal positive weight-related outcomes, but the changes measured were small and the measures utilized varied among the studies [19]. Other school-based interventions report varying degrees of success, with modest changes in behavior paired with mixed results with obesity indicators [17,19]. However, considering the global reach of childhood obesity, past reviews have failed to hone in on the differences between approaches taken internationally and within the United States. It is imperative health professionals continue to build on previous lessons learned. Yet, we cannot assume a one-size-fits-all approach will work across such diverse populations. Thus, this systematic review hopes to bridge the gap between the previous major systematic reviews of its kind in an effort to gain a better understanding of global initiatives aiming to target the critical public health dilemma childhood obesity and overweight pose [20]. Therefore, the purpose of this review is to compare and contrast U.S. and international school-based obesity prevention interventions and highlight efficacious strategies to aid development of future interventions.

## 2. Experimental Section

### 2.1. Methods

#### Inclusion/Exclusion Criteria

Inclusion criteria for this review included: (1) primary research; (2) an overweight or obesity prevention intervention; (3) school-based; (4) peer-reviewed and published between 1 January 2002 through 31 December 2013 in selected databases; (5) available in the English language; (6) a child-based program, which could include parents; and (7) outcome-based. Exclusion criteria were: (1) interventions implemented in preschools, early childcare programs, or after-school programs; (2) not available in the English language; (3) obesity treatment interventions (*i*.*e*., only focused on an obese population); and (4) articles reporting study design and/or process evaluation only. In this review, primary research was defined as studies which were carried out to acquire data first-hand, rather than being gathered from previously published sources. In addition, school-based was operationalized as an intervention that was implemented during regular school hours for children in kindergarten through senior year of high school. Interventions that took place outside of regular school hours, both before and after school, were excluded from this study.

### 2.2. Rationale for Review

This systematic review is an update of existing reviews and incorporates childhood obesity interventions implemented worldwide. The Community Guide conducted an extensive review published in 2005, covering the years of 1966 through 2001. However, this review resulted in only ten studies and included both school and work-based settings [19]. The Cochrane Database System Review was also similar, though it covered school, community, clinic, and family-based programs and included pre-school aged children, spanning years 1990 through February 2005 [21]. Similarly, two school-based obesity prevention reviews were published in 2006 and 2007 [12,18]. However, the first was limited to those interventions conducted outside of the United States and the second only included interventions within the United States and the United Kingdom. Although there is overlap regarding dates of inclusion with these reviews, this systematic review includes interventions within the United States and worldwide.

### 2.3. Study Abstraction

**Figure 1:**
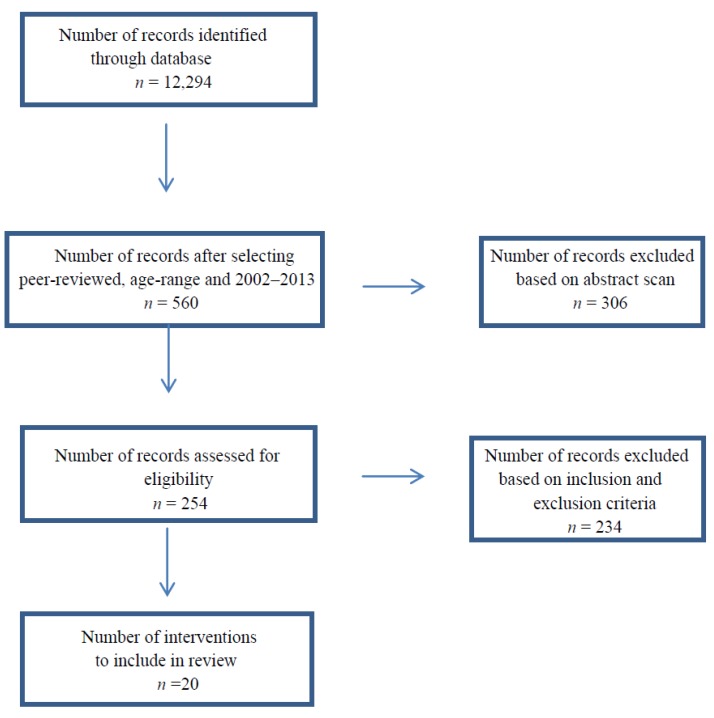
A summary of search results.

Two researchers conducted an extensive literature search in order to incorporate all pertinent studies in this review. Searches were conducted utilizing the following databases: Academic Search Premier, Cumulative Index to Nursing and Allied Health (CINAHL), Medical Literature Analysis and Retrieval System Online (MEDLINE), Education Resources Information Center (ERIC), and Psychology and Behavioral Sciences Collection. The following keywords were used: [obese OR overweight] AND [school OR school-based] AND [youth OR child OR adolescent] AND [prevention OR intervention OR treatment OR program OR study]. Limits of scholarly journals (peer-reviewed) were set. Initially, 12,294 articles were originally identified using the keywords. Articles were then further reduced based on inclusion and exclusion criteria. See Figure 1 for a flow diagram summary of the search results. Twenty interventions fulfilled the criteria and are included in this review.

### 2.4. Data Extraction

Data from the studies were extracted, independently, by two researchers using a standardized form that the researchers created. Discrepancies were examined and final non-disputed data recorded. Extracted data included: author, year of publication, participant data, theory used, research design, outcomes, intervention dosage and duration, strategies utilized, and attrition and follow-up rates.

## 3. Results and Discussion

### 3.1. Results

The included studies have been summarized in Table 1 and Table 2, outlining the target population, intervention strategies and design, measures, outcomes, and relevant findings. The interventions have been divided into two sections, United States and international, and arranged alphabetically within sections.

**Table 1:** Summary of included interventions—sample, design, intervention description.

Author, Year, Reference #	Sample Description	Sample Size	Research Design	Dosage and Duration	Intervention Strategies
**United States**					
Chehab *et al*. 2007 [22]	U.S.; HS; Low-income; BL 49% OW; 28% OB	*N =* 46 girls	Pre-experimental	Weekly 2-h sessions; 29 weeks	Homework, aerobic activity, sampling of healthy foods, cooking exercises, group recitation of motivational catch phrases
DeVault *et al*. 2009 [23]	U.S.; ELEM; Low-income; BL BMI not assessed	*N =* 140; Tx. = 71, Cnt. = 69	Quasi-experimental	Six 30-min. weekly lessons; 6 weeks	Fruit and vegetable bingo, baking whole-grain bread to bring home, comparing portion sizes of snack foods
Donnelly *et al*. 2009 [24]	U.S.; ELEM; low-income; BL BMI Int. 17.9 +/− 3.1; Cnt. 18.0 +/−3.7	*N =* 1527; Cnt. = 713, Tx. = 814	RCT	90 min/week of physically active academic lessons; 3 years	EI; Existing lessons from Take 10!^®^, PA incorporated across all content areas
Foster *et al*. 2008 [25]	U.S.; Low-income; BL 17% OW, 22–25% OB	*N =* 1349; Tx. = 749, Cnt. = 600	RCT	50 h/year; 2 years	EI; PC; School self-assessment, nutrition education, nutrition policy, social marketing
Hollar *et al*. 2010 [26]	U.S.; ELEM, Low-income; 7.3% Tx. OW; 8.5% Cnt. OW; 17.6% Tx. OB; 22.9% Cnt. OB	*N =* 1173; Tx. = 974, Cnt. = 199	Quasi-experimental	Monthly nutritional activities, 10–15 min. PA/day, & structured activities during PE; 2 years	EI; incorporated nutritious ingredients and whole foods, provided a healthy lifestyle curricula, hands on school-based wellness activities such as gardens
Johnston *et al*. 2013 [27]	U.S.; ELEM; BL 33% OW/OB	*N =* 835; PFI (professional facilitated information): *N =* 509 SH (self-help): *N =* 326	RCT	5 teaching moments/week, 1 lesson/week, 1 activity/2 weeks, and 1 school-wide activity/semester; 2 years	EI; PC; Healthy messages and lessons were applied to all subject areas
Manger *et al*. 2012 [28]	U.S.; ELEM; BL Cnt. OW 21%; Tx. OW 15%; Cnt. OB 14%; Tx. OB 14%	*N =* 697; Tx. = 396, Cnt. = 301	Quasi-experimental	8 weekly lessons, 30 min. each; 2 years	PC; Food charts and games, hula hoops and skip ropes, songs to promote healthy eating
Melnyk *et al*. 2009 [29]	U.S.; HS; BL Mean BMI percentile 80.5 Tx.; 71.33 Cnt.	*N =* 19; Tx. = 12, Cnt. = 7	RCT	2–3 times/week; 9 weeks	Educational information on leading a healthy lifestyle, role-playing, participation in group PA wearing pedometers.
Pbert *et al*. 2013 [30]	U.S.; HS; Low-income; BL 78.6% Int. OB; 60% Cnt. OB	*N =* 82; Tx. = 42, Cnt. = 40	RCT	6 one-on-one sessions; 2 months	5-3-2-1-0 approach to support making 5 key behavior changes
Wang *et al*. 2010 [31]	U.S.; ELEM; Low-income; BL: OW/OB not assessed	*N =* 327	Prospective	Integrated daily; 2 years	EI; PC; Change in school food, school dining, offering of cooking classes, school gardens, lesson integration, food diaries
**International**					
Graf *et al*. 2008 [32]	International; ELEM; BL: OW 8.1%; OB 6.6%	*N =* 615	Quasi-experimental	One extra health lesson/week (20–30 min.), and one 5 min. PA break/morning; 4 years	PC; health lessons, mini PA breaks
Hartmann *et al*. 2010 [33]	International; ELEM; OW And/or OB: 1st grade Cnt. 25%; 1st grade Tx. 26%; 5th grade Cnt. 26%; 5th grade Tx. 25%	*N =* 411; 1st grade Cnt.=69; 1st grade Tc.p:=111; 5th grade Cnt.=85; 5th grade Tx. *p =* 146	RCT	Daily PE, short activity breaks/day during lessons, PA homework playground changes; 1 year	EI;PC; Increased PA, playground changes, PA homework
James *et al*. 2004 [34]	International; ELM; BL: 27.6% F Tx. OW; 20.1% M Tx. OW; 5.7% F Tx. OB; 4.1% M Tx. OB; 28% F Cnt. OW; 18.8% M Cnt. OW.; 7.3% F Cnt. OB; 1.7% M Cnt. OB	Tx. =15 clusters, *N =* 325. Cnt. = 14 clusters, *N =* 319	RCT	Four 1 h sessions; 1 year	EI; Educational sessions, drink diary
Kanyamee *et al*. 2013 [35]	International; ELEM; Low-income; Mean BMI z scores Tx. = 2.39 (*SD = 0*.42); Cnt.=2.53 (*SD* = 0.56)	*N =* 136; 68 per group	RCT	Weekly; 18 weeks	Dietary intake recorded daily, computer games, cartoon animation, and comic books
cLlargues *et al*. 2011 [36]	International; ELEM; BL 16.7% Cnt. OW; 20.3% Tx. OW; 18.1% Cnt. OB; 9.6% Tx. OB	*N =* 509; Tx. = 272, Cnt. = 237	RCT	3 h/week; 2 years	EI; PC; CR; Regular PA, hands on activities like cooking workshops and promotion of playground games.
Lopes *et al*. 2009 [37]	International;ELEM; BL BMI mean and s.d. in girls 6–7: 16.4 +/− 3, girls age 8 and +: 17.7 +/− 3.6. Boys 6–7: 16.8 +/− 2.6 and boys 8 and +: 17.8 +/− 3.2	*N =* 168; 81 from one school and 87 from another	Quasi-experimental	30 min/day; 2 weeks	EI; access to extra exercise/play equipment
Muckelbauer *et al*. 2009 [38]	International; ELEM; low-income; BL OW Tx. 23.4%; Cnt. 25.9%	*N =* 2950; Tx. = 1641, Cnt. = 1309	RCT	Daily (water fountain exposure), four 45-min. classroom lessons; 1 year	EI; installment of water fountains, distribution of water bottles, associated classroom lessons
Sachetti *et al*. 2013 [39]	International; ELEM; BL Cnt. OW 24.1%; Tx. 25% OW; Cnt. 8.8% OB; Tx. 10.4% OB.	*N =* 428; Tx. = 212, Cnt. = 216	RCT	30 min PA, two 50 min. sessions/week of extra PE/week; 2 years	EI; School yard & classroom activity including circuits, games, exercises
Walther *et al*. 2009 [40]	International; ELEM; BL BMI percentile Cnt. 52.5 +/− 28.8; Int. 50.5 +/− 28.9.	*N =* 188; Tx. = 112, Cnt. = 76	RCT	Int. = 45 min. PA/day. Cnt. = 45 min. exercise twice/week, healthy lifestyles session once/ month; 1 year	EI; Increased PA, lessons on lifestyles
Wong & Cheng, 2013 [41]	International; ELEM; BL 100% OW/OB	*N =* 185; MI (*n* =70) ; MI + group (*n =* 66); Cnt. = 49	Quasi-experimental	14-week, six-section program. 30-min./session; 11 months.	PC; Diet journal, exercise log, motivational interviewing

Table Key: BL=Baseline; CR= Culturally relevant; EI= Environmental Influence; ELEM=Elementary; OB = Obese; OW = Overweight; PA=Physical activity; PC= Parental Component.

**Table 2:** Summary of interventions—outcomes, measures, salient findings.

Author, Year	Primary Outcome (s)	Measures	Measures-Time	Attrition	Salient Findings
**United States**					
Chehab *et al*. 2007 [22]	BMI	Ht., wt.	Baseline, 29 weeks	84.8% completed all components	For OB & OW girls, positive relationship (*p* < 0.01) between wt. loss and extent of program participation
DeVault *et al*. 2009 [23]	Nutrition-related knowledge, attitudes, behaviors	Surveys	Baseline, 6 weeks, 3 weeks follow up	46% Cnt. & 54% of the Tx. completed both surveys all three times	Behavioral intent for food choice sig. increased at post- for Int. *vs*. Cnt. *p* < 0.014
Donnelly *et al*. 2009 [24]	BMI	Ht., wt., academic achievement	Baseline, 3 years	2.5% dropped out	Change for overweight to at-risk approached significance (*p =* 0.08).
Foster *et al*. 2008 [25]	OW, OB	Ht., wt. dietary intake, PA, sedentary behavior	Baseline, spring of year 1, 2 years	Int. & Cnt. schools at 1 (31.9% *vs*. 31.5%) & 2 years (36.0% *vs*. 39.2%)	Cnt. = 15%, Int. = 7.5% overweight in 2 years. After controlling for gender, race/ethnicity, age, predicted ORI of overweight were ~33% for the Int. group (OR = 0.67; 95% CI: 0.47–0.96; *p* < 0.05)
Hollar *et al*. 2010 [26]	BMI	Ht., wt., FCAT scores	Baseline, 2 years	Not mentioned	Decrease BMI between baseline and post-intervention: Cnt.: OW = 6.8 %, Tx. = 2.1% (*p =* .27)
Johnston *et al*. 2013 [27]	zBMI, academic outcomes	Ht., wt., year-end final grades, GPA	Baseline, 24 months	79% completed all msmnts.	Students who were OW/OB in the PFI sig. reduced (zBMI) compared to SH group (*p* < 0.001).
Manger *et al*. 2012 [28]	BMI	Ht., wt.	Baseline, annually	The final data set included 697 students, 125 of whom had 2 and 572 of whom had 3 assessments of BMI	Adjusted Mean BMI % declined from 66.1 to 65.0 in Cnt., 62.8 to 58.9 in Int. (*p* = 0.015)
Melnyk *et al*. 2009 [29]	Triglycerides, lipoproteins, beliefs, nutritional knowledge, depressive symptoms	Ht., wt., BMI, waist circum., blood work, student- completed evaluations	Baseline, post-intervention	89% provided complete baseline and post-intervention data	Tx.: increased commitment to make healthy choices (via choices scale)—at baseline: 54.5 and post-intervention: 58.91 (*p* = 0.07)
Pbert *et al*. 2013 [30]	BMI, waist circum., percent body fat, BP	Ht., wt., BMI, BP, waist circum., dietary intake, PA via accelerometer	Baseline, 2 months, 6 months	100% remained	Adjusted Mean change BMI 6 months post 95% CI (*p* < 0.676) Cnt.: = 0.23 (−0.46, 0.910, Tx. = 0.01 (−0.66, 0.68)
Wang *et al*. 2010 [31]	Nutrition knowledge, fruits & vegetables	Surveys, food diaries, interviews with teachers & administration	Food behavior assessed annually, surveys completed by students (not specified)	82.3% remained	Students most exposed to intervention increased fruits & vegetables by 0.2 cups, students least exposed decreased 0.3 cups (*p* < 0.05)
**International**					
Graf *et al*. 2008 [32]	Endurance, motor, coordination tests	Ht., wt., BMI, motor tests, body coordination tests	Baseline, end of second school year, end of fourth school year	2% dropped out	23.2% (13/56) of OB and OW children from the Tx. reached normal weight at final exam
Hartmann *et al*. 2010 [33]	Physical, psychosocial QOL	QOL (survey), pubertal stages, anthropometry, body composition, sociodemographic variables	Baseline, 1 year	90% had valid post-intervention data (*N =* 411)	PA had sig. effect on psychosocial QOL in OW (*p* < 0.05) and urban first graders (*p* < 0.05)
James *et al*. 2004 [34]	Drink consumption, OW, OB	Ht., wt., waist circum., BMI	Baseline, 6 months, 1 year	89% remained at 1year	12 months post, Mean %> than 91st percentile for BMI Cnt. = 26.9%., Tx. = 20.1%
Kanyamee *et al*. 2013 [35]	Intention to perform eating behaviors, eating behaviors, BMI	Intention to perform eating behavior for wt. control; eating behaviors for wt. control, ht., wt., BMI	Baseline, 6 weeks, 18 weeks	100% remained	At 18 weeks, Mean BMI for age (z scores) *p* < 0.001—Tx. = 2.00, Cnt.: = 2.55
Llargues *et al*. 2011 [36]	BMI	Changes in eating habits, PA	Baseline, 2 years	Complete data obtained 72.3%, Cnt. = 237 (78.8%), Tx. = 272 (72.7%)	Cnt.: OW = 24.9%, OB = 10.7%. Tx.: OW = 25.1%, OB = 8.9%, *p* < 0.001
Lopes, Lopes, and Pereira, 2009 [37]	PA levels	Gender, age, BMI accelerometer	Baseline, 2-weeks	24 students were excluded	Sig. effects for total PA (*p*<0.001). Sig. interaction between gender & age (*p =* 0.009)
Muckelbauer *et al*. 2009 [38]	BMI	Ht., wt., gender, age, migrational background, survey	Baseline, 1 year	92% completed intervention	BMI SDS changes from baseline to follow-up assessment were 0.005 +/− 0.289 in the Tx. & 0.007 +/− 0.295 in the Cnt.
Sachetti *et al*. 2013 [39]	PA habits, physical performances, and BMI	Ht., wt., BMI, motor tests	Baseline, 2 years	14.2% and 13.9% in Tx. and Cnt. groups did not complete	Decrease (boys: 10%; girls: 12%) in daily sedentary activities, *p* < 0.05; Int. lower rise in BMI compared to the Cnt. (*p* < 0.001)
Walther *et al*. 2009 [40]	BMI-SDS, leukocyte msmt., HDL, motor quotient score	Body composition, BP, HR, body coordination, spirometry. Blood work, survey	Baseline, 1 year	3 were lost in follow up for both the Cnt. and Int. groups (6 total)	Decrease OW and OB in Tx. from 12.8% to 7.3%
Wong & Cheng, 2013 [41]	Change in wt.-for-ht. %	Changes in weight-related behaviors, anthropometric measures	From the 4th to the 11th month after baseline	4 did not complete, not specified as to which group	Sig. increase in the avg. calories consumed due to increase in PA in past 7 days in MI group (*p* < 0.01) and MI+ group (*p* < 0.01). Sig. change at post in BMI, fat %, anthropometric measures

#### 3.1.1. Sample and Design

The review was limited to interventions that took place in a school-based environment. Of the interventions, half (*n =* 10) took place outside of the United States [32,33,34,35,36,37,38,39,40,41] while the remaining half (*n =* 10) were implemented in the United States. Overall, the majority (85%) of interventions took place at the elementary school level [23,24,25,26,27,28,31,32,33,34,35,36,37,38,39,40,41] and 15% (*n =* 3) took place at the high school level [22,29,30]. However, no international interventions targeted high school populations and none of the interventions focused on middle school settings. More U.S. interventions indicated they specifically targeted low-income schools (70%, *n =* 7) compared to international interventions (30%, *n =* 3). The number of participants within the interventions varied significantly, ranging from 19 to 2950 participants. Overall, the average number of participants in the interventions implemented in the United States (*M* *=* 619.5) was higher compared to those implemented internationally (*M* = 591.5), but both varied immensely in terms of range.

Study design reported throughout the interventions included 60% (*n =* 12) [24,25,29,30,31,33,34,35,36,38,39,40] randomized controlled trials, in which participants were randomly assigned to control or intervention groups. Seven international interventions compared to five interventions implemented in the United States incorporated use of a randomized controlled trial. Six of the interventions (30%) were quasi-experimental [23,26,28,32,37,41] in which participants were not randomly assigned to a control or comparison group. One study was of a pre-experimental design [22] and one study incorporated a prospective research design [31], both of which were implemented in the United States.

#### 3.1.2. Theoretical Framework

Use of theory was only mentioned in 30% (*n =* 6) of the interventions [29,30,32,35,36,38], four of which were implemented internationally. The most commonly used theory was the Theory of Planned Behavior (*n =* 3, 14.3%) [32,35,38]. Other theories mentioned included: Cognitive Behavioral Theory, Social Cognitive Theory, the Precaution Adoption Process Model, and Investigations, Vision, Action, Change (IVAC). Of the interventions that did utilize a theory and/or model, four of the six studies detailed how the theory was operationalized [29,30,35,36].

#### 3.1.3. Intervention Approach

Duration of the interventions ranged from two weeks (*n =* 1) [37] to four years (*n =* 1) [32]. Interventions lasting one year or less comprised 55% (*n =* 11) of those included in this review. The remaining studies, 45% (*n =* 9), lasted two years or more, six of which were implemented in the United States. Dosage of the interventions varied from daily to weekly sessions. Of the included interventions, 45% (*n =* 9) took place weekly or more than once throughout the week, while 35% *n =* 7) of the interventions took place daily. Additionally, 20% of the studies (*n =* 4) did not specify dosage, reporting only that the intervention was administered throughout the total duration of the implementation period.

A variety of strategies were utilized throughout the included interventions. Strategies included providing and/or implementing educational sessions, cooking classes, school-based gardens, sampling healthy foods, change in school food options, and promotion of physical activity. Almost half of the interventions, 45% (*n =* 9) [22,23,26,31,34,35,36,41], explicitly mentioned incorporating hands-on nutritional activities such as school gardens, journaling of food intake, participating in cooking classes, sampling healthy foods, and playing games such as “fruit and vegetable bingo.” Hands-on nutrition activities were implemented in five United States *vs*. four international interventions. Increasing the amount of physical activity was another common strategy overall (40%, *n =* 8) [22,23,24,28,33,37,39,40], more so with interventions implemented internationally (*n =* 5). Twelve of the interventions (60%) involved some form of environmental change(s) [24,25,26,27,31,33,34,36,37,38,39,40], seven of which were implemented internationally. Environmental changes ranged from increasing the required time allotted daily for physical education to modification of nutritional offerings. Eight of the interventions (40%) included a parental component [25,27,28,31,32,33,36,41], with varying degrees of involvement ranging from parental phone calls, meetings, and information and instructions sent home to reinforce information given at schools. Parental involvement was similar when comparing international and United States interventions.

Over half (57%) of the programs were implemented by a classroom teacher. Others implementing the interventions included physical education teachers, researchers, research assistants, school nurses, support staff, occupational therapists, physicians, and nutritionists. One quarter of the interventions mentioned the use of incentives [22,25,30,31,38]. Among the incentives used were a reusable water bottle, weekly raffle prizes, a group television appearance on “The Today Show,” $25 gift certificates, and raffle tickets for physical activity-related prizes.

#### 3.1.4. Intervention Outcomes and Measures

All of the interventions provided outcome data, although the primary outcome varied. BMI was the primary outcome for the majority of interventions (55%, *n =* 11). In addition, the following primary outcomes were mentioned: academics (*n =* 2) [26,27], cholesterol levels (*n =* 2) [29,40], physical activity habits (*n =* 2) [37,39], incidence of overweight/obesity (distinctive from BMI) (*n =* 2) [25,34], and nutrition knowledge (*n =* 2) [29,31].

Likewise all of the interventions reported positive changes throughout the interventions related to at least one of their identified primary outcomes, as summarized in Table 2. Successful outcomes included positive changes in endurance and coordination tests, physical activity levels, depressive and anxiety symptoms, triglycerides, lipoproteins, physical and psychological quality of life, HDL cholesterol, leukocyte measurement, intention to change and improvement in eating behaviors, and water consumption. Overall, 70% of the interventions reported a decrease in BMI and/or overweight or obesity. More international interventions (*n =* 8) compared to those implemented in the United States (*n =* 6) reported this as a significant outcome.

Of the 20 interventions, 30% (*n =* 6) [23,27,32,38,39,41] followed-up with participants post-intervention, but the findings from such follow-ups varied. Of the five interventions that explicitly mentioned the follow-up results [23,32,38,39,41], three noted positive findings, including significantly lower increases in BMI and/or risk of overweight [38,39], improved food self-efficacy, enhanced perception of one’s own body image, and attempted weight loss [23]. Additionally, one intervention resulted in a significant increase in anthropometric data as a result of growth [31] and another showed no significant difference in BMI between the control and intervention groups at a six-month follow-up [41].

### 3.2. Discussion

Given the concern of childhood obesity [1,2,4] and the consequential health-related outcomes and societal impact worldwide [9,10,11], the purpose of this review was to compare and contrast U.S. and international school-based obesity prevention interventions and highlight efficacious strategies.Overall, findings are promising, considering each of the global school-based interventions included in this review resulted in at least one positive, measurable outcome. The majority of interventions, both international and in the United States, took place at the elementary school level. Elementary schools appear to be an ideal setting for childhood obesity prevention interventions given the vast array of opportunities for promoting physical activity and nutrition education through practice, policy, and supportive environments [12]. Targeting specific grades and classrooms within elementary schools may be easier when compared to targeting middle schools and high schools due to scheduling, built in opportunities for physical activity, and flexibility in the curriculum. This may very well help to explain a more marked stabilization in childhood obesity rates across developed nations among this age group [5]. Interestingly, no international interventions were implemented in high school settings and none, either in the United States or internationally, were implemented in middle schools. There is a need for future obesity prevention interventions to target these at risk groups, particularly considering the lack of decline and/or plateau in recent obesity prevalence among young and older adolescents, and the likelihood that the health behaviors and associated risk will continue into adulthood [9].

A critical component of successful school-based obesity prevention interventions is tailoring the program to the targeted audience [16]. Incorporating formative research prior to intervention implementation may assist with these efforts and thereby enhance sustainability and the likelihood of positive outcomes [35]. This seems to be particularly important when working in schools with a high prevalence of low-income children. Seventy percent of the interventions implemented in the United States indicated they specifically targeted low-income schools compared to only 30% internationally. This is, perhaps, explained by the focus on reducing health disparities in the United States and the resulting funding initiatives typically giving precedent to those interventions which target low-income, at-risk populations. It is encouraging that intervention outcomes among low-income children were found to simultaneously improve academic performance and weight status [41]. Given the determinants of obesity linked to socioeconomic status [7,12], future consideration needs to be given to this population. In developing countries, there is support that the ‘adiposity gap’ between low-income, middleclass, and upper class is widening [5]. Therefore, other developing countries may deem it necessary to also target low-income, high-risk groups as cost considerations are taken into account [5].

There were several commonalities and distinct differences when contrasting approaches used with interventions implemented internationally and in the United States. All of the interventions focused on promoting physical activity and/or healthy food behaviors. Interventions implemented in the United States tended to integrate more hands on nutrition activities, whereas international interventions were more likely to incorporate promotion of physical activity as an intervention strategy. Interventions that utilize both a physical activity and nutrition component may increase the effectiveness of school-based childhood obesity prevention programs [17]. However, the majority of interventions included in this review did not take such an approach. Future interventions should build on successful nutrition and physical activity strategies as part of a multi-pronged approach [16]. Yet, feasibility needs to be considered, as some schools may not have the resources necessary to implement strategies targeting nutrition and physical activity simultaneously. A tiered, stage-based plan can be put into place so efficacious school-based strategies are strengthened each year of implementation. This type of approach will enhance sustainability and likelihood of long-term impact [16] and may be more appealing to schools considering obesity prevention interventions.

Parental influence with regards to children’s nutrition and physical activity behaviors is a well known determinant of childhood obesity [12,14,15,16]. However, only 40% of the included interventions incorporated parental involvement as a targeted strategy. Of those that involved parents throughout the intervention, 75% reported significant weight and/or BMI reductions, warranting support for this strategy as part of school-based programs aiming to prevent childhood obesity. While the role that schools can play in childhood obesity interventions is important, it is also important to consider the crucial role that parents play in implementing, encouraging, and reinforcing healthy behaviors. Research indicates the cooperation of parents in addressing physical activity and nutrition concerns in children is essential [21]. Incorporating motivational interviewing as a component of parental education seems to be a beneficial strategy [41] along with requesting parents to complete food diaries to determine change in child efficacy and parental involvement [31]. Future research should determine the feasibility of such approaches as well as the potential positive health outcomes garnered, not only with the child, but also with the parents and related family members. Considering the lack of parental involvement was similar across interventions implemented in the United States and internationally, there is a need to further explore cultural barriers as to why this might be the case.

Another intervention strategy worth mentioning is the integration of some form of environmental change. Over half of the interventions incorporated at least one environmental change, seven of which were implemented internationally. Significant changes in BMI and/or weight outcomes were reported in 75% of the interventions that included an environmental component, giving support to include as an impactful strategy. Environmental strategies seem to have become a focus with more recent interventions, as represented by the included studies, with all but one being implemented after 2008. Consequently, evaluation of such efforts needs to be conducted to determine the most efficacious school-based strategies. Although there is a push for policy change related to obesity prevention efforts in the United States [16], it is clear that the United States can build on lessons learned from the successful environmental strategies incorporated in several of the included international interventions.

Incorporating evidence-based strategies is critical to increase the likelihood of successful school-based obesity prevention interventions. However, equally important is the use of theory when designing intervention programs, as evidence suggests they are more effective compared to interventions that do not utilize a theory [17]. Of the interventions included in this review, less than one third incorporated a behavioral theory, with the TPB occurring most frequently. The applicability of TPB for childhood obesity interventions is difficult to determine, given the small number of interventions reporting use of this theory and even fewer fully describing how the theory was operationalized. Without such information, it is difficult to make comparisons across interventions. Future research should explore integration of behavioral theories and their related theoretical constructs to measure intervention outcomes. This may aid in a researcher’s ability to prioritize intervention strategies and consequently replicate these successful strategies [42].

Although difficult to compare outcomes due to the varying nature of the intervention design, target population, and selected primary outcomes, study findings demonstrate the potential for school-based obesity prevention interventions. Overall, 70% of the interventions reported a decrease in BMI and/or overweight or obesity, with BMI serving as the most common primary outcome. More international interventions compared to those implemented in the United States reported these significant outcomes. This is somewhat conflicting with recent research indicating an increased likelihood of significant anthropometric changes in populations with higher incidence of obesity [5]. The rates of obesity in the United States continue to be greater as compared to other developed countries [5], so one would expect the potential for significant changes in BMI and overweight/obesity to be higher, even in general population school-based obesity prevention interventions. Given this fact, there is a need to further investigate factors impacting intervention outcomes.

Comparing the most impactful school-based interventions, the following commonalities existed, regardless of whether they were implemented internationally or in the United States: (1) large sample size; (2) elementary school setting; (3) weekly or daily dosage; (4) duration of one year or greater; (5) inclusion of environmental strategy; and (6) multi-pronged approach. Table 3 summarizes recommendations for future school-based obesity prevention interventions.

**Table 3:** Recommendations for school-based obesity prevention interventions.

Strength of research design should be considered. Theoretical framework should assist program development and implementation. Intervention should be tailored to target audience (*i*.*e*., low-income, grade level, geographic location). Integrate a combination of nutrition and physical activity strategies. Parents play a crucial role in childhood behavior change and should be included. Environmental strategies should be considered. Teachers are most likely to assist with program implementation and should receive adequate training. Intervention duration of one year correlates with positive BMI outcomes and sustainability. Incorporate multiple outcomes, including knowledge, attitudes, behaviors, related theoretical constructs, and anthropometric data.Implement follow-up measures to determine long-term efficacy of the intervention.

One important benefit of school-based obesity prevention interventions not yet mentioned is minimal attrition throughout the implementation period. Schools provide a captive audience for the majority of the year. Two interventions [30,35] reported 100% of participants completing the duration of the intervention, with lengths of two to four months. Although attrition rates tended to increase the longer the intervention duration, the majority of participants completed all measures. Interventions with a duration of one year and BMI as a primary outcome resulted in an average of 92% completion—lending support for a long-term intervention. Attrition in school-based interventions can often not be helped, particularly when students move to a new school, consequently making it difficult to follow-up. Therefore, the reasons participants did not complete the interventions were often not reported in the included school-based studies. Future research should aim to differentiate attrition categories to include those students who moved to another school and/or other extenuating reasons. This will aid feasible intervention design and development of appropriate follow-up measures. Additionally, international interventions tended to have higher attrition rates compared to their U.S. counterparts. As a result, the methodology of international interventions should be more closely studied to determine if the higher rates of attrition are related to cultural influences, efficacy of program implementation, or other factors. Also, follow-up of any kind was infrequent throughout studies and its value should be considered in future interventions, given the need to determine lasting efficacy.

## 4. Limitations

Limitations within this review should be noted. As this is a qualitative review, data were collected, examined, and summarized in a narrative format; a quantitative meta-analysis was not performed. For that reason, all study designs were included. Although the majority of the interventions incorporated a RCT or quasi-experimental design, this is important to mention. Furthermore, studies were omitted if they were not in English, which could limit the applicability of this review to other countries also suffering from a childhood overweight and obesity epidemic. Studies that were conducted outside of regular school hours were excluded considering differences in potential intervention strategies and target population. In addition, interventions that failed to report one or more outcomes were excluded as it was not possible to evaluate the efficacy of such studies. Studies that were published prior to 1 January 2002 were excluded, but many were included in a previous comprehensive review—justifying the reasoning to limit studies published after that date. Only peer-reviewed articles within the included databases were included, increasing the likelihood of publication bias, since unpublished studies were not reported. Although childhood obesity is a popular topic and an abundance of articles were retrieved from the initial search, every effort was made to be comprehensive and systematic throughout data collection.

## 5. Conclusions

With 43 million children globally considered overweight or obese, 92 million at risk of becoming overweight, and a projected increase of childhood overweight and obesity estimated to reach 9.1% worldwide in 2020, childhood obesity has become a public health crisis in dire need of support [4]. School-based interventions are essential in the fight against global childhood obesity since many children lack nutrition and/or physical activity education, resources, and support outside of their homes [12,13,14,15,16]. As supported by the promising outcomes reported in this review, childhood obesity can be mitigated through the use of school-based interventions. Given the number of hours per day that children spend in schools, they afford an excellent medium through which to implement obesity prevention interventions. Although differences did exist when comparing interventions implemented in the United States and internationally, common themes emerged which should be shared widely with health professionals and school personnel planning and implementing school-based obesity prevention programs. There is a need to incorporate multi-pronged, tailored strategies in interventions with duration of one year or more. Environmental changes also seem to be promising as impactful intervention strategies. In addition, there is a need to include extensive follow-up measures to assess the long-term efficacy of school-based interventions [21]. There is no need to reinvent the wheel when designing similar obesity prevention programs. Health professionals need to work together to share lessons learned in order to promote cost-effective and efficacious school-based interventions that will impact the childhood obesity epidemic. The continued support, implementation, and monitoring of these types of evidence-based programs will help to combat the rising rates of childhood obesity worldwide.
